# Parameter Estimations of Dynamic Energy Budget (DEB) Model over the Life History of a Key Antarctic Species: The Antarctic Sea Star *Odontaster validus* Koehler, 1906

**DOI:** 10.1371/journal.pone.0140078

**Published:** 2015-10-09

**Authors:** Antonio Agüera, Marie Collard, Quentin Jossart, Camille Moreau, Bruno Danis

**Affiliations:** Laboratoire de Biologie Marine CP160/15, Universite Libre de Bruxelles, F. D. Roosevelt, 50, 1050 Brussels, Belgium; The Evergreen State College, UNITED STATES

## Abstract

Marine organisms in Antarctica are adapted to an extreme ecosystem including extremely stable temperatures and strong seasonality due to changes in day length. It is now largely accepted that Southern Ocean organisms are particularly vulnerable to global warming with some regions already being challenged by a rapid increase of temperature. Climate change affects both the physical and biotic components of marine ecosystems and will have an impact on the distribution and population dynamics of Antarctic marine organisms. To predict and assess the effect of climate change on marine ecosystems a more comprehensive knowledge of the life history and physiology of key species is urgently needed. In this study we estimate the Dynamic Energy Budget (DEB) model parameters for key benthic Antarctic species the sea star *Odontaster validus* using available information from literature and experiments. The DEB theory is unique in capturing the metabolic processes of an organism through its entire life cycle as a function of temperature and food availability. The DEB model allows for the inclusion of the different life history stages, and thus, becomes a tool that can be used to model lifetime feeding, growth, reproduction, and their responses to changes in biotic and abiotic conditions. The DEB model presented here includes the estimation of reproduction handling rules for the development of simultaneous oocyte cohorts within the gonad. Additionally it links the DEB model reserves to the pyloric caeca an organ whose function has long been ascribed to energy storage. Model parameters described a slowed down metabolism of long living animals that mature slowly. *O*. *validus* has a large reserve that—matching low maintenance costs- allow withstanding long periods of starvation. Gonad development is continuous and individual cohorts developed within the gonads grow in biomass following a power function of the age of the cohort. The DEB model developed here for *O*. *validus* allowed us to increase our knowledge on the ecophysiology of this species, providing new insights on the role of food availability and temperature on its life cycle and reproduction strategy.

## Introduction

Antarctic marine environments include some of the most extreme ecosystems on Earth [1]. Marine organisms in Antarctica are adapted to extremely low but stable seawater temperatures. In fact, high latitudes in Antarctica are probably among the most stable regions of Earth in terms of the lack of seasonal variability in temperature [2]. In contrast, strong seasonality in day length results in large variations of ice cover and phytoplankton biomass [3]. Adaptation to such an environment has resulted in organisms with a poor capacity to survive elevated temperatures [4] yet capable of surviving long-term low-food availability [5]. These adaptations are of concern within the context of global climate change. It is now largely accepted that the Southern Ocean is particularly vulnerable to global warming with some regions already being challenged by rapid rates of temperature rise [6,7]. Global warming has a cascading effect that is causing a variety of changes in Antarctic marine ecosystems including a decrease in the duration and extent of seasonal sea ice, an increase in seasonal floor ice scouring or local decreases in salinity due to glacial melt [7,8]. Climate change is influencing both physical and biotic components of marine ecosystems, and will have an impact on the distribution and population dynamics of Antarctic marine organisms. Life history, distribution and abundance of species reflect the operation of metabolic processes in the context of varying environments [9]. Knowledge of metabolic processes is essential to understanding a species population dynamics, performance, and functional role within a given ecosystem. In order to better delineate the potential effects of climate change on Antarctic benthic marine ecosystems, a more comprehensive knowledge of the life history and physiology of key species is urgently needed [10].

The Southern Ocean’s remoteness represents a logistical challenge, complicating the study of biodiversity and ecosystem dynamics. Despite considerable research efforts invested over recent decades (under the auspices of international programmes such as the Census of Antarctic Marine Life, http://www.coml.org/projects/census-antarctic-marine-life-caml), our knowledge of Antarctic marine organisms remains limited. Only a handful of species have been relatively well studied, whereas our understanding of the vast majority of species is limited to documenting their presence, with little baseline information on their biology, habitat requirements, or population dynamics [1,11]. This handful of well-studied species is primarily represented by near shore shallow-water species, which are abundant, easily accessible from field stations and readily collectable using trawls, baited traps or SCUBA, and easy to maintain in experimental set-ups. However even for these well studied species our knowledge is limited by their own unique biology (e.g.: slow metabolism), as well as the characteristics of their environment (e.g. depth, seasonal sea ice). In Antarctic waters, ectotherms are generally very slow growing due to a constant low temperature and prolonged periods of limited food resources [1,5]. Similarly, they often display adaptations in reproduction such as gametogeneic cycles that can span several years [12–14], and slow development of larvae that may be released into the water column or brooded [15,16]. Evidence suggests that Antarctic benthic organisms are vulnerable to high-mortality events due to environmental stressors such as warming, salinity change, ocean acidification, ice scour, disease outbreak, pollution or overfishing [17,18].

The development of the Dynamic Energy Budget (DEB) theory provided first-principles models describing the processes of energy and matter-uptake and their use for maintenance, development, growth and reproduction [19,20]. DEB theory is unique in capturing the metabolic processes of an organism through its entire life cycle as a function of temperature and food availability [20]. This allows the integration of a species’ physiology with its physical environment. DEB theory is a mechanistic theory that resulted in the DEB model [20]. DEB theory can model the underlying physiological processes based on first principles (e.g. mass-energy conservation laws, linkage of processes to volume or surface, homeostasis of compounds) [21] that are common to all life forms. Moreover, the model allows for the inclusion of the different life history stages, and thus, becomes a tool that can be used to model lifetime feeding, growth, reproduction, and their responses to changes in biotic and abiotic conditions [22,23]. The DEB model provides a timely opportunity to model the effects of environmental changes on the population dynamics of key Antarctic marine species at a juncture when global climate change is rapidly altering polar ecosystems [10].

In the present study we estimated the parameters of the DEB model for an abundant Antarctic species: the sea star *Odontaster validus* (Koehler, 1906). *O*. *validus* has a circumpolar distribution, being one of the most abundant and conspicuous benthic animal in Antarctic shallow waters [24]. *O*. *validus* is a generalist predator, a filter feeder and a scavenger, feeding on a wide range of food resources [5]. Its omnivorous diet as well as its high abundance qualifie *O*. *validus* as a major species that plays an important role in nutrient and energy flow within Antarctic shallow-water benthic ecosystems. Due to its conspicuous nature, *O*. *validus* was one of the first Antarctic marine species to be studied in shallow water, and its ready availability soon made it a model for the study of Antarctic marine invertebrates [24]. A number of studies have focused on diverse aspects of the biology and ecology of *O*. *validus*, including aspects of its growth, reproduction, energetics, and the potential effects of climate warming and ocean acidification; many of which encompass components of both adult and larval life histories e.g.: [12,25–31]. However, several knowledge gaps in the life history of this common species persist, including immediately post- settlement growth, age at first reproduction, fecundity, life-span, and the effect of food and temperature on all these parameters, among others. *O*. *validus* is a highly successful species that inhabits areas with contrasting availability in food resources, seasonal ice and subject to ice scouring, making it particularly well suited as a model organism for the application of DEB models to describe its life history and its interaction with the environment.

The aim of the present study was to use DEB theory to mechanistically describe the entire life cycle of *O*. *validus*, considering both larval and adult stages. This study provides a quantitative physiological model that can be used to better understand the physiological condition of *O*. *validus*, and its interactions with its ecosystem in response to a growing need to model the effects of changes in the physical environmental on the population dynamics of key species. A DEB model will be able to fill some of the existing gaps in the life history of *O*. *validus* including age at first reproduction, fecundity and the role of temperature and food variability in its life cycle and condition. The generality of DEB models will facilitate comparisons of *O*. *validus* with closely related temperate and tropical species to discern possible adaptations of the energy budget in an Antarctic species.

## Materials and Methods

### Model description

**Fig 1:**
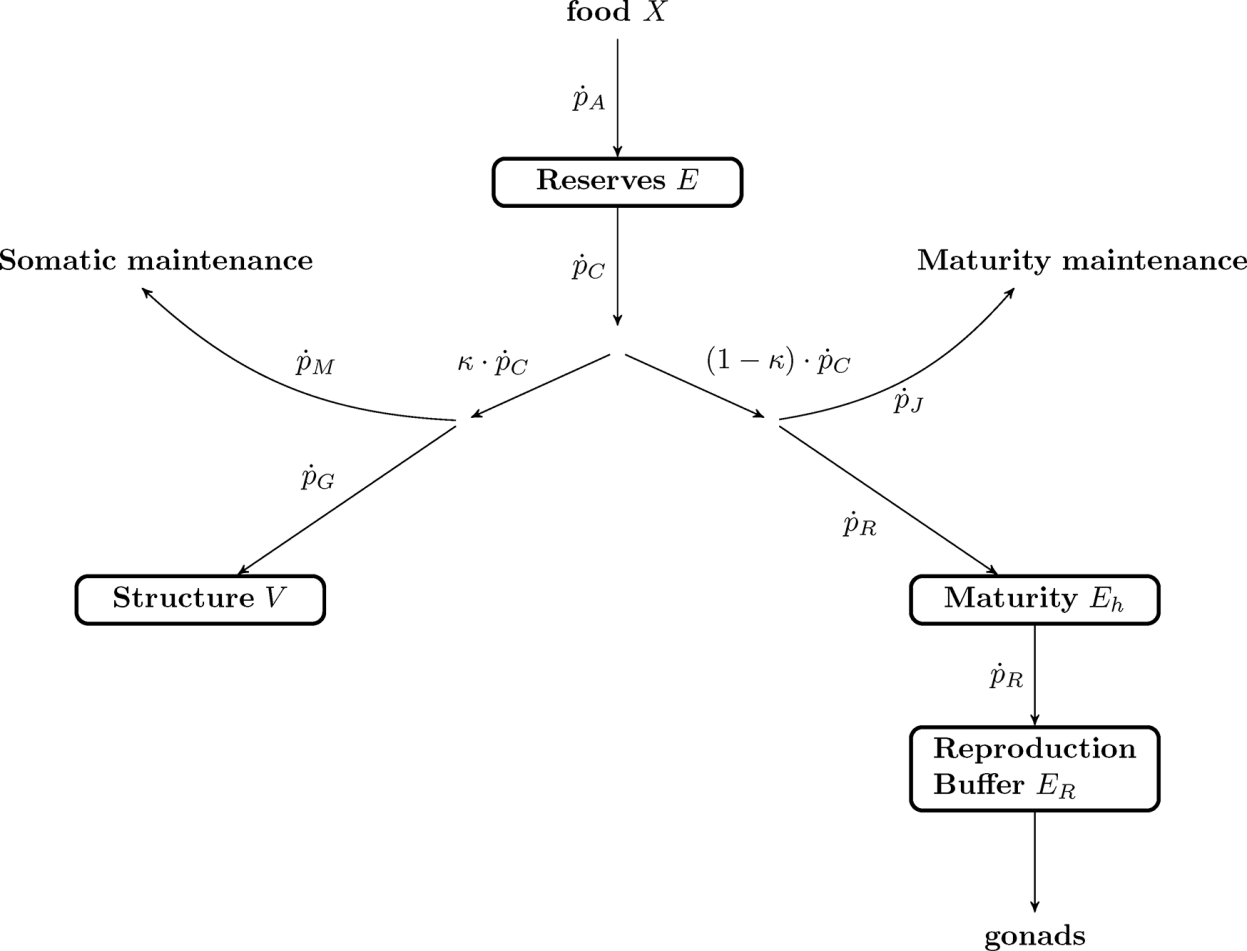
Schematic representation of the standard DEB model [18]. Arrows represent energy fluxes (J d^-1^) that drive the dynamics of the four state variables (boxes). Energy enters the organism as food (*X*), is assimilated at a rate of ${\dot{p}}_{A}$ into reserves (*E*). The mobilization rate (${\dot{p}}_{C}$), regulates the energy leaving the reserve to cover somatic maintenance (${\dot{p}}_{M}$), structural growth (${\dot{p}}_{G}$), maturity maintenance (${\dot{p}}_{J}$), maturation (${\dot{p}}_{R}$) (immature individuals) and reproduction (${\dot{p}}_{R}$) (mature individuals). *κ* is the proportion of the mobilized energy diverted to ${\dot{p}}_{M}$ and ${\dot{p}}_{G}$, while the rest is used for ${\dot{p}}_{J}$ and ${\dot{p}}_{R}$.

Dynamic Energy Budget (DEB) theory describes the energy and mass flows of an organism throughout its life history [20,21]. The DEB model tracks an organism’s energy flow between four state variables: reserve (*E*), structural volume (*V*), maturation (*E*_*H*_) and reproduction buffer (*E*_*R*_) (Fig 1). Energy enters the organism as food (*X*) and is assimilated at a rate of ${\dot{p}}_{A}$ into reserves. The mobilization rate (${\dot{p}}_{C}$) regulates the energy leaving the reserves to cover somatic maintenance (${\dot{p}}_{M}$), structural growth (${\dot{p}}_{G}$), maturity maintenance (${\dot{p}}_{J}$), maturation (${\dot{p}}_{R}$) (immature individuals) and reproduction (${\dot{p}}_{R}$) (mature individuals). *κ* is the proportion of the mobilized energy diverted to ${\dot{p}}_{M}$ and ${\dot{p}}_{G}$, while the rest is used for ${\dot{p}}_{J}$ and ${\dot{p}}_{R}$. In DEB, assimilation is a function of available food, following a functional response of Hollings’ type II. Mobilization however depends on the amount of energy in the reserves (Fig 1, see S1 Appendix for detailed description of DEB assumptions and explanation of DEB notation)

Like many other echinoderms and representative of other marine phyla, *O*. *validus* undergoes pelagic larval development and metamorphosis before reaching its final adult stage. Several DEB model parameters are related to structural volume or surface. Structural dimensions are related to measurable dimensions through a shape coefficient and as such the standard DEB assumes isomorphism (animal shape does not change with growth). Therefore the relation between physical size and structural volume and surface is the same as the animal grows. Additionally, DEB model intends to describe the entire life cycle with the same set of parameters. However, during larval development, *O*. *validus* has a different larval body shape (feeding bipinnaria larva followed by a feeding brachiolaria larva) and grows exponentially until metamorphosis [31]. Exponential growth relates to metabolism acceleration that results from a change in the size-specific feeding, assimilation (${\dot{p}}_{A}$) and mobilization (${\dot{p}}_{C}$) [32]. In the present study the standard DEB model was modified to incorporate these relevant aspects of the life history of sea stars: changes involved the use of a different shape coefficient (*δ*_*M*_) and an “acceleration” factor (*S*_*M*_). Therefore, the present DEB model considers two different shape coefficients, one for the larvae (*δ*_*M*.*larv*_) before metamorphosis, and another for the juvenile and the adult that follow metamorphosis (*δ*_*M*_). The acceleration factor accounts for the changes in the parameters related to the exponential growth period between the moment the larva is able to feed (birth) and metamorphosis [20,32].

### Estimation of DEB model parameters

DEB models are based on observations of natural populations and experimental studies where the effects of controlled variables (e.g. food availability or temperature) on feeding, growth and metabolic rates of individuals are measured. There is a robust literature on various aspects of the life history, biology and ecology of *O*. *validus*. In the present study we extracted literature-based data for parameter estimation (see Table 1 for a parameter list and corresponding definitions). A large part of the data were sourced from a long-term (2 yr) experiment conducted by J. S. Pearse and I. Bosch at McMurdo Station, Ross Sea (77°51’ S; 166°40’ E) between 1984 and 1986, whose methods can be found in [27]. These data provide essential information on growth rates and reproduction from individuals of *O*. *validus* fed *ad libitum* at constant temperature. We combine laboratory data with field observations McMurdo Station. Details on additional data sources are provided in the context of their use below.

**Table 1:** DEB parameters values for *Odontaster validus*. These parameters are given for a temperature of 285 K.

Parameter	Symbol	Value±SD	Units
**Basic DEB Parameters**			
**Maximum surface area-specific assimilation rate** ^1^	*{* ${\dot{p}}_{Am}$ *}*	6.82 ±2.42	J d^-1^ cm^-2^
**Volume-specific cost of maintenance** ^2^	*[* ${\dot{p}}_{M}$ *]*	6.93±1.91	J d^-1^ cm^-3^
**Volume-specific cost of structure** ^1^	*[E*_*G*_ *]*	2579±549	J cm^-3^
**Fraction of energy allocated to somatic maintenance and growth** ^1^	*κ*	0.81±0.03	-
**Scaled functional response at McMurdo** ^1^	*f*_*f*_	0.80±0.17	-
**DEB compound parameters**			
**Energy conductance** ^1^	$\dot{\nu }$	0.007±0.001	cm d^-1^
**Maturity maintenance rate coefficient** ^1^	$\dot{k_{J}}$	0.001±0.02	d^-1^
**Shape coefficients**			
**Post-metamorphic** ^2^	*δ*_*M*_	0.57±0.02	-
**Pre-metamorphic** ^1^	*δ*_*M*.*lrv*_	0.59±0.71	-
**Temperature sensitivity**			
**Arrhenius temperature** ^3^	*T*_*A*_	5303±86.81	K
**Lower limit of tolerance range** ^3^	*T*_*L*_	269.5±23.8	K
**Upper limit of tolerance range** ^3^	*T*_*H*_	288±0.29	K
**Arrhenius temperature at lower limit** ^3^	*T*_*AL*_	120200±10^6^	K
**Arrhenius temperature at upper limit** ^3^	*T*_*AH*_	68360±24430	K
**Conversion parameters**			
**Density of structure** ^4^	*d*_*V*_	1	g cm^-3^
**Weight-energy coupler for reserves** ^4^	*ρ*_*E*_	4.35x10^-5^	g J^-1^
**Molecular weight of reserves** ^4^	*w*_*E*_	23.9	g mol^-1^
**Chemical potential of reserves** ^4^	${\bar{\mu }}_{E}$	550	kJ mol^-1^

^1^ Estimated using the covariation method

^2^ Estimated from data and adjusted with covariation method

^3^ Estimated by non-linear least squares

^4^ Fixed [20]

A series of DEB model parameters can be estimated directly from experimental studies and field observations [21,33] as follows:

#### Sensitivity to temperature

DEB theory uses the Arrhenius concept of enzyme activation to incorporate the effect of temperature on the metabolic processes. Arrhenius temperature (*T*_*A*_) provides information on the variation of metabolic rates with temperature and can be calculated from observed values of metabolic rate measured at different temperatures. The DEB model uses a curve for temperature sensitivity given by 5 parameters (S1 Appendix) These five parameters were estimated using oxygen consumption rates at different temperatures (data from Peck et al. 2008). Values were scaled by dividing by the maximum observed oxygen consumption rate. Parameters were obtained by adjusting the Arrhenius function (equation (10) in S1 Appendix) to scaled values by means of a nonlinear least squares regression using package minpack.lm [34] (function *nlsLM*) in R v.3.1 [35]. This method required starting values for the parameters: for *T*_*A*_ the starting parameter was calculated by the linear regression: log(scaled rate) = a + *T*_*A*_ . (1/K) between 271.5 and 285 K. The lowest and maximum observed temperatures were used as starting values for *T*_*L*_ and *T*_*H*_. *T*_*AL*_ and *T*_*AH*_ starting values were those calculated for the sea star *Pisaster ochraceus* [22]. As the inflexion point of the data was not clear (there were only two points after 285K) *T*_*1*_ (reference temperature) was also free and using a starting value of 285 K.

#### Post-metamorphic shape coefficient

A post-metamorphic shape coefficient (*δ*_*M*_) was calculated based on the relationship between the body wet weight (*W*_*w*_) and the animal’s arm length (measured from the middle of the oral disc to the tip of an arm) (*L*_*w*_) by fitting the equation *W*_*W*_ = (*δ*_*M*_ . *L*_*w*_)^3^ by means of a weighted least squares regression.

#### Volume-specific maintenance cost

Volume-specific maintenance cost [${\dot{p}}_{M}$] was approximated from oxygen consumption of starved individuals at 12°C [29], following the approach used by van de Veer et al. [33] for the bivalve *Mytilus edulis*. The values obtained are likely including other processes besides structural maintenance, as starvation was probably not long enough to completely deplete the reserves, however the value was related to the actual *[* ${\dot{p}}_{M}$ *]* and considerably lower than that calculated for *Pisaster ochraceus* [22] (even after correction for temperature), and as such a good starting value for the covariation method. Although starting values should not influence final estimation, the quality of those improve the estimation process and increase the success of parameter estimation [36,37].

We used the covariation method for further estimation of the DEB model parameters [37,38], estimated with MATLAB® 2014b using the software package DEBtool (available at http://www.bio.vu.nl/thb/deb/deblab/debtool/). The covariation method links the parameters to experimental and field observations of different life stages and approximates the parameters using a Nelder-Mead numerical optimization to minimize the difference between observed and predicted values based on a weighted least-squares criterion [38].

The parameters previously approximated were used as starting values in the covariation method. The parameters without experimental estimation comprised pseudo-data and their starting values were yielded from DEB theory [39,40] and closely related species. DEB theory is formulated with the premise that all organisms regulate metabolism using the same mechanisms, meaning that differences among organisms are mainly due to different parameter values affecting the common mechanisms. The covariation method is then completed with direct observations and data yielded from experiments for which it will approximate the parameters. There are two different types of observations that can be used in the covariation method [37]: zero-variate data are single data points for a range of different physiological observations; uni-variate data comprise a list of paired numbers where one number is an independent variable (e.g. time, temperature) and the other is a dependent variable (e.g. mass, oxygen consumption). The goodness of fit of the covariation method is given by the mean absolute relative error (MRE) [36] among all data points and sets used in the parameter estimation. The procedure also outputs MRE for each of the uni-variate variables to help assess the goodness of fit of predictions to each data set. A list of the zero-variate data used in the estimation of the DEB model parameters can be found in Table 2, alongside the references from which the data was sourced. Uni-variate data are represented in the results with references when the data are used.

**Table 2:** Zero-variate data used for the estimation of the DEB model parameters. All values are given at a temperature of 271.5 K. This is the temperature at which these values where measured. MRE: mean absolute relative error.

Variable		Obs	Pred	Units	MRE	Reference
Age at birth^1^	a_b_	33	27	d	0.19	[31]
Age at metamorphosis^2^	aj	165	162	d	0.02	[31]
Length at birth^1^	L_b_	0.04	0.03	cm	0.24	[31]
Length at metamorphosis^2^	L_j_	0.14	0.16	cm	0.17	[31]
Length at puberty^3^	L_p_	2	2.15	cm	0.08	[26]
Maximum length^4^	L_i_	7	7.10	cm	0.01	Pearse et al. *Unp*. *data*
Maximum length at *f*_*f*_ ^5^	L_i_	6	5.63	cm	0.05	[25]
Egg dry weight	Wd_0_	1.1	1.18	μg	0.07	[46]
Weight at puberty^3^	W_p_	2.95	2.84	g	0.04	[26]
Maximum weight^4^	W_i_	100	105	g	0.05	Pearse et al. *Unp*. *data*
Maximum weight at *f*_*f*_ ^5^	W_i_	50	49.5	g	0.01	[25]
Gonadosomatic Index^6^	GSI	0.10	0.08	-	0.16	Pearse et al. *Unp*. *Data*
Pyloric Index^7^	PI	0.30	0.30	-	0.00	Pearse et al. *Unp*. *Data*

^1^ birth is set at the moment the animal starts or is able to feed.

^2^ moment at which the brachiolaria larvae is ready to settle for metamorphosis

^3^ start of first gametogenesis

^4^ maximum size reached by the species when there is no food limitation

^5^ observations of maximum size at McMurdo field station

^6^ maximum gonad index for an animal of the maximum size, gonad index as gonad weight/total wet weight.\]

^7^ pyloric index for the food conditions in the laboratory as pyloric caeca weight/(total wet weight–gonad weight)

There was no data available on the functional response of *O*. *validus*. Moreover, *O*. *validus* is omnivorous and will likely exhibit a different functional response towards different food items. However, in DEB models the scaled functional response can be yielded from the individuals’ condition as *f* is in equilibrium with the available resources, providing information of the energy intake and individual condition. Using the covariation method and data on length-weight and maximum size of the *O*. *validus* population at McMurdo, we approximated the scaled functional response for this population (*f*_*f*_) relative to the scaled functional response of the animals fed in the lab for which a scaled functional response of *f* = 1 was set.

DEB theory assumes that the state variables (*E*, *V*, *E*_*H*_, *E*_*R*_) are not directly measurable, and could not be related to a particular body component. Linking state variables to measurable body components is valuable for the application of DEB models in population dynamics models and an effort has been made towards this, for example *E*_*R*_ is now generally linked to gonad tissue [33,39]. In sea stars the pyloric caeca has traditionally been linked to energy reserves [41,42] as this body component comprises almost all the energetic reserve compounds that can be found in sea stars [43] (besides gonads). Moreover, previous studies showed that this organ is linked to environmental resource availability [42,44], as such it is probably related to the DEB energy pool (*E*, Fig 1, S1 Appendix). A preliminary parameter estimation using the covariation method already provided a good approximation of the pyloric caeca organ index, and therefore this organ index (PI; weight pyloric caeca / (total weight–gonad weight)) and the pyloric caeca weight at size were used in the final parameter estimation.

### Reproduction buffer handling rules

*Odontaster validus* is dioecious with external fertilization of spawned gametes occurring in the water column. Reproduction takes place once a year with spawning occurring over several months [12,27]. While *O*. *validus* reproduces once a year, gametogenesis lasts for time periods ranging from 18 to 24 months, at least for females [12]. This reproduction strategy involves the simultaneous maturation of two different cohorts of oocytes separated by a year. These cohorts are easily differentiated within the ovary as they mature at different rates. Pearse [12] provides a detailed description of oogenesis in *O*.*validus*.

**Fig 2:**
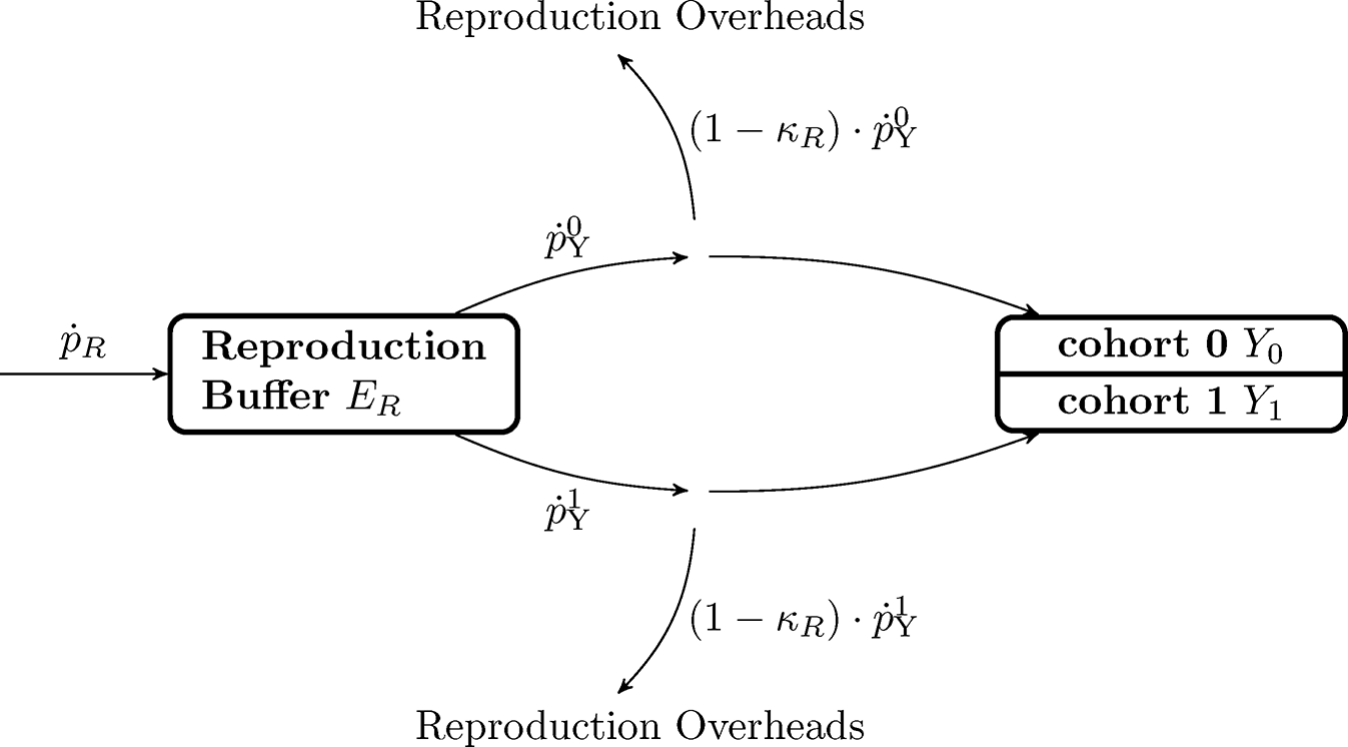
Schematic representation of the reproduction buffer handling rules for *Odontaster validus*. Arrows represent energy fluxes (J d^-1^). After paying for maturity costs ${\dot{p}}_{R}$ is stored in the reproduction buffer *E*_*R*_ that is continuously being mobilized to develop the gonads. The gonads contain two different cohorts of oocytes, each one receiving energy at a rate given by ${\dot{p}}_{Y}^{n}$, however cohort 1 is 365 days older than cohort 0 and ${\dot{p}}_{Y}^{n}$ depends on *t* (age). *κ*_*R*_ is the reproduction efficiency and represent the energy dissipated as the buffer is transformed into eggs.

In DEB models, investment in reproduction is calculated from the mobilization flux ${\dot{p}}_{C}$ when the individual becomes a reproductive adult. This energy is stored in the reproduction buffer *E*_*R*_ to be used later for the maturation of gonads and production of eggs or sperm (Fig 1). However, the standard DEB model does not include any handling rules for how the reproduction buffer is transformed into gonads, nor does it provide any information about what triggers the maturation of gonads and reproduction [20]. To improve the applicability of the DEB model for population dynamics we established handling rules for the reproduction buffer [45]. The first assumption is that energy allocated to the reproduction buffer is constantly being transformed into gonad; oogenesis is taking place constantly in *O*. *validus*. When allocated to the gonads, the energy is split asymmetrically among the two cohorts of oocytes, which results in the older cohort growing faster (Fig 2). The detailed data on the oogenesis of *O*. *validus* provided by the comprehensive study of Pearse [12] facilitated the development of a precise rule for how *O*. *validus* handles gonadal development, and the respective contribution of the different cohorts of gametes present. The changes in the relative volumes of each oocyte cohort during development [12] suggest that each cohort receives resources more or less continuously. We assumed that oogenesis within each cohort always follows the same pattern and that each cohort is independent of the others presence within the ovary. We also assumed that only two cohorts are developing at any time. While there may be a third cohort of oocytes present, the latter cohort is composed by fully developed oocytes being spawned. We assumed that a new cohort starts developing in June, as is the case for the population of *O*. *validus* near McMurdo Station [12], and that the cohort’s development lasts exactly two years. Once mature, the cohort remains within the gonad until spawning begins in July, after which oocytes are released following a linear decrease until October.

With the previous considerations and using data on oocyte growth and abundance in the gonad [12], we were able to estimate the proportional weight of a oocyte cohort at all times (considering also the changes in the gonad size due to the development of other cohorts and spawning). The energy allocated to each cohort (${\dot{p}}_{Y}^{n}$) as it develops was best described as a function of the power of its age (*t*, time in days since the onset of development for that cohort):

$${\dot{p}}_{Y}^{n}\;=\;{\dot{p}}_{R}\cdot a\cdot t^{b}$$ (1)

We linked the cohort development to the DEB allocation to the reproduction buffer (${\dot{p}}_{R}$). In that manner the growth of the gonad will also depend on the size of the individual, the environmental temperature, and food availability. In order to do so, we estimated the parameters *a* and *b* as a function of the proportion of the mobilization flux (${\dot{p}}_{C}$) allocated to reproduction (${\dot{p}}_{R}$) after paying maintenance costs for maturity (${\dot{p}}_{J}$):

$${\dot{p}}_{R}\;=\;(1-\kappa )\cdot {\dot{p}}_{C}-{\dot{p}}_{J}$$ (2)

Before being transformed into gonads, part of ${\dot{p}}_{Y}^{n}$ is dissipated as reproduction overheads (*κ*_*R*_ = 0.95; reproduction efficiency, fixed value [20,22]) related to inefficiencies of product transformation. ${\dot{p}}_{Y}^{n}$ also needs to be converted from energy (J d^-1^) into weight (g d^-1^) using the weight-energy coupler for reserves (*ρ*_*E*_). We assumed that energy reserves and gonad have a similar composition. In that manner the growth rate in weight of each cohort of oocytes is given by:

$$\frac{dY_{n}}{dt}\;=\;\rho_{E}\cdot \kappa_{R}\cdot {\dot{p}}_{Y}^{n}$$ (3)

We calculated the values for *a* and *b* in (3) using a non-linear least squares regression (package minpack.lm [34] function nlsLM in R v.3.1 [35]) and the DEB parameters previously determined using the covariation method.

### Model exploration

We explored the model performance by plotting weight from the time of fertilization until reaching the maximum size given by DEB parameters. Further exploration was done by comparing model prediction of GSI (gonadosomatic index, gonad weight/ total weight) and PI (pyloric index, pyloric caeca weight/(total weight–gonad weight)) with field and laboratory data not used during the parameter estimation sourced from Pearse & Bosch experiment [27] and McClintock et al. [25] observations.

## Results

### DEB parameters calculated from empirical data

**Fig 3:**
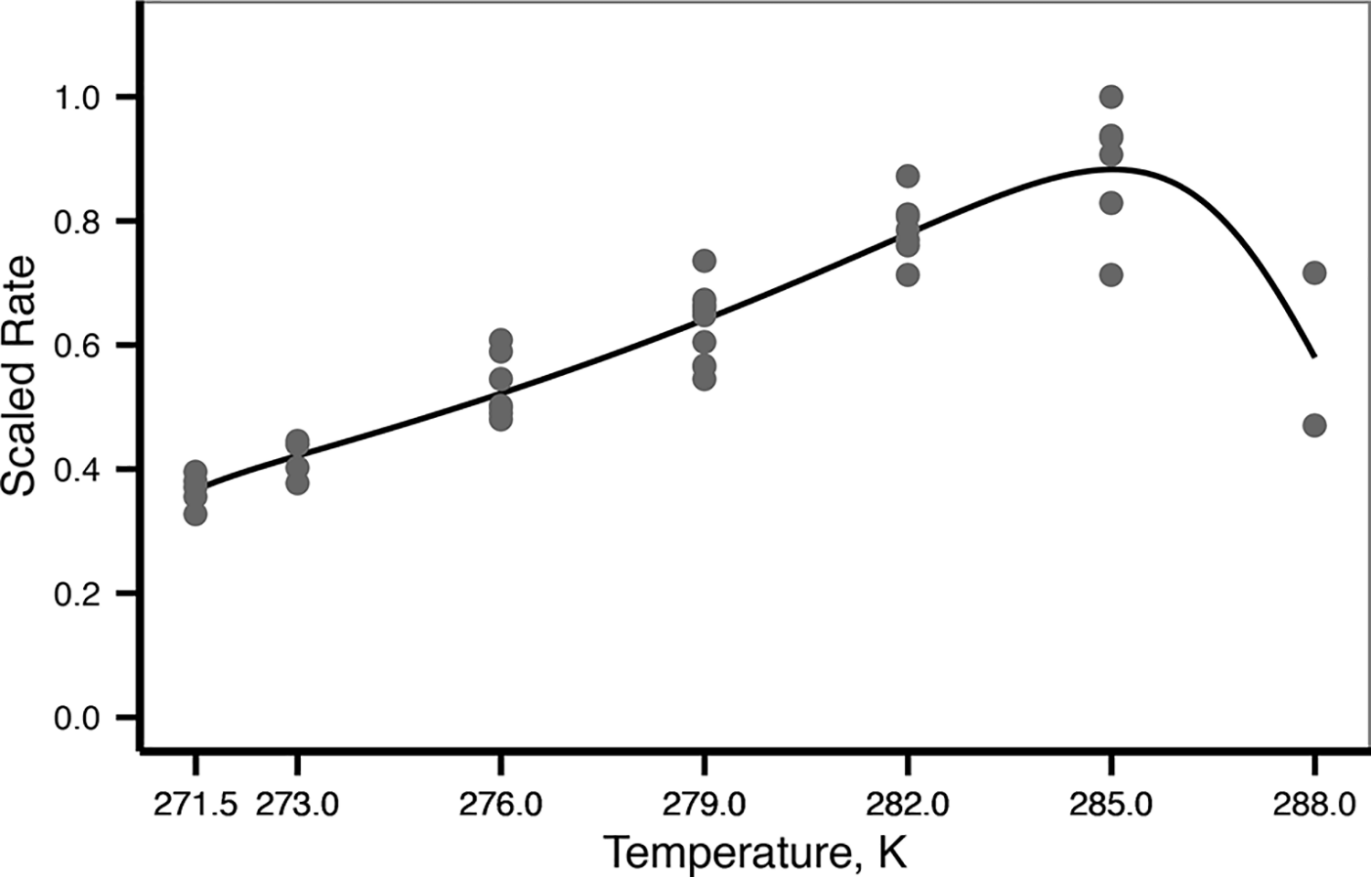
Temperature sensitivity. Dots are the scaled values of observed oxygen consumption rates. The line represents the adjusted Arrhenius function (parameters are in Table 1).

Adjusting the Arrhenius function to the scaled respiration rates provided by Peck et al. [29] resulted in a *T*_*A*_ of 5166 K, and a maximum tolerance limit of 288 K (AIC = -109, MRE: 0.065, see Table 1 for the other parameters values, Fig 3). These values and the other Arrhenius function parameters were used as fixed values in the covariation method to determine the other DEB parameters.

The post-metamorphic shape coefficient was calculated from observations of individuals kept in captivity over a year at McMurdo Station (Pearse et al., *unpublished data*), and yielded a value of 0.63±0.07. This value was optimized later using the covariation method (final value in Table 1). The data on oxygen consumption of starved individuals by Peck et al. [29] allowed an approximation of the value of the volume-specific cost of maintenance *[* ${\dot{p}}_{M}$ *]* using the approach described by van der Veer et al. [33]. Following a starvation over a period of four weeks, the oxygen consumption by *O*. *validus* at 285 K was on average of 558 μg d^-1^ cm^-3^, which resulted in an energetic cost of 7.564 J d^-1^ cm^-3^. This value was also optimized during parameter estimation using the covariation method (final value in Table 1. Parameters are all given at 285 K, the maximum oxygen consumption was yielded at this temperature [29].

### Estimation of DEB parameters using the covariation method

**Fig 4:**
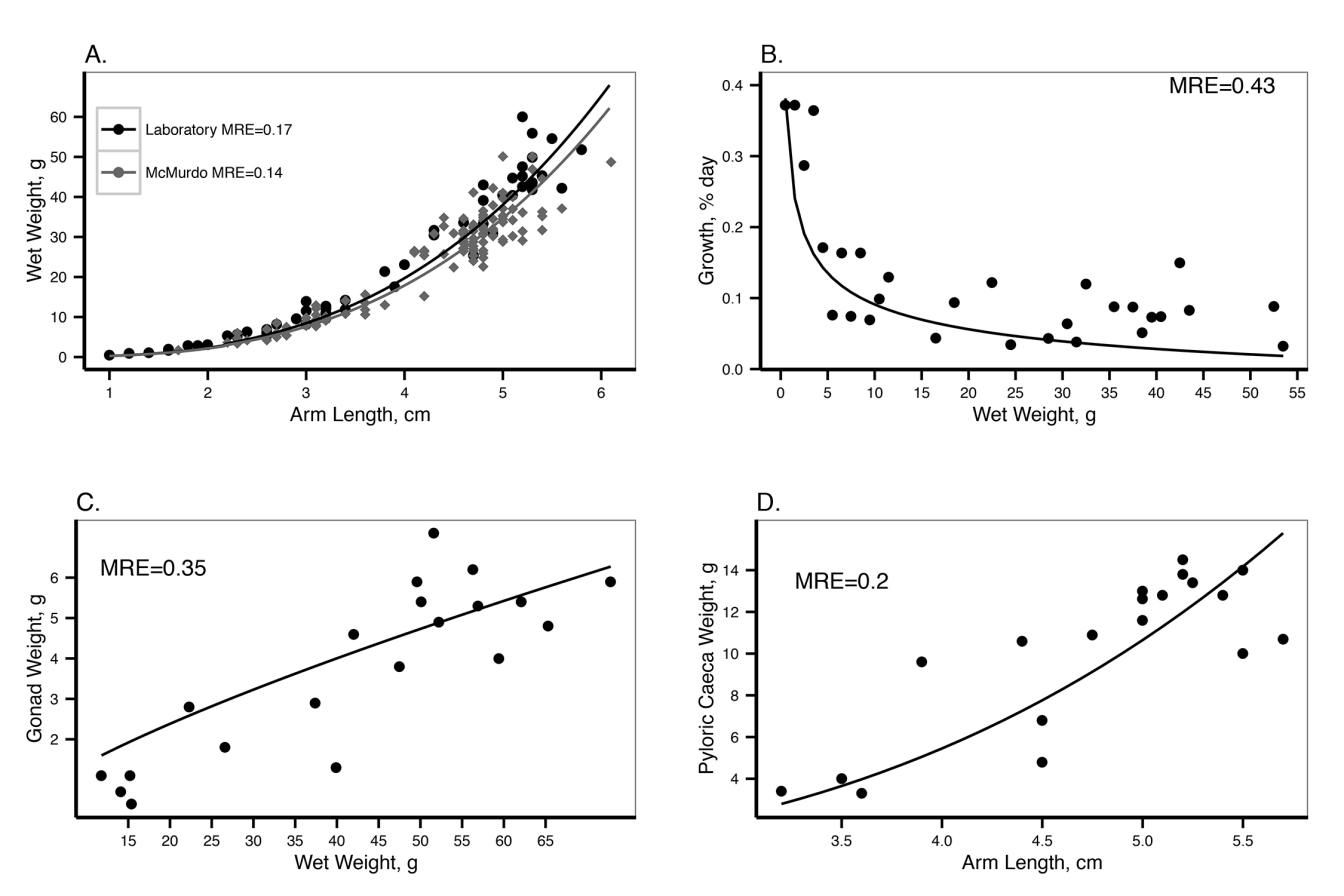
DEB model output with the data on post-metamorphic individuals used for parameter estimation. A. Length-weight relationship, dots are data from laboratory experiments, fed *ad libitum* (Pearse et al. *unpublished data*), diamonds are values from McMurdo station at the same time of the year [23]. B. Proportional growth rate by day according to size. Dots are data from laboratory experiments, animals fed *ad libitum* [23]. C. Maximum gonad weight according to size. Dots are observed data from laboratory experiments, animals fed *ad libitum* (Pearse et al. *unpublished data*). Maximum gonad size is reached before reproduction takes places around June in McMurdo [12]. D. Pyloric caeca weight according to size. Dots are observed data from laboratory experiments after being fed *ad libitum* for more than a year (Pearse et al. *unpublished data*). MRE is the mean absolute relative error.

**Fig 5:**
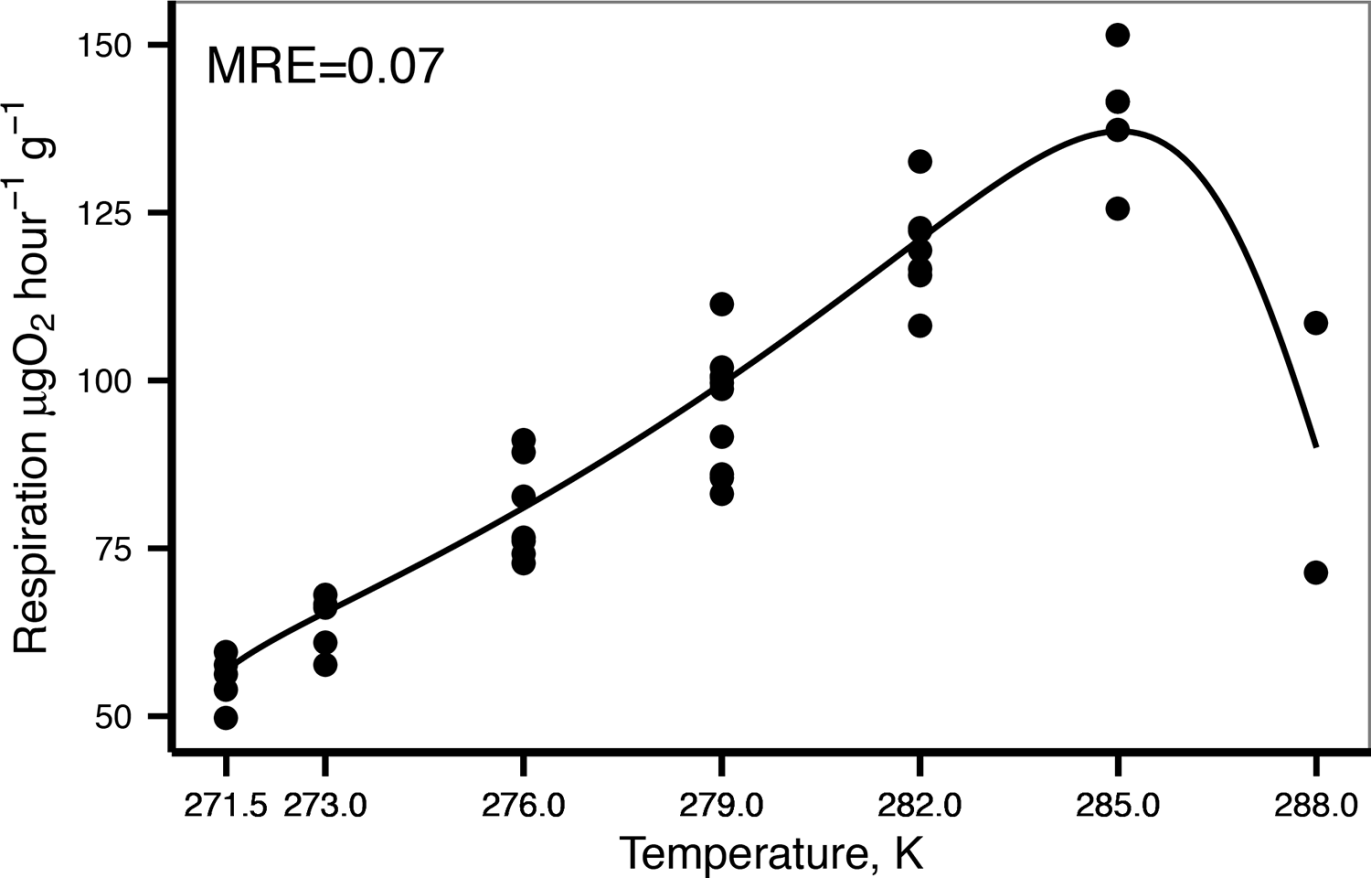
Oxygen consumption according to temperature. Dots are observed values for several individuals averaging 7.2 g wet weight [27]. The line represent DEB model predictions using the estimated values from Table 1. MRE is the mean absolute relative error.

**Fig 6:**
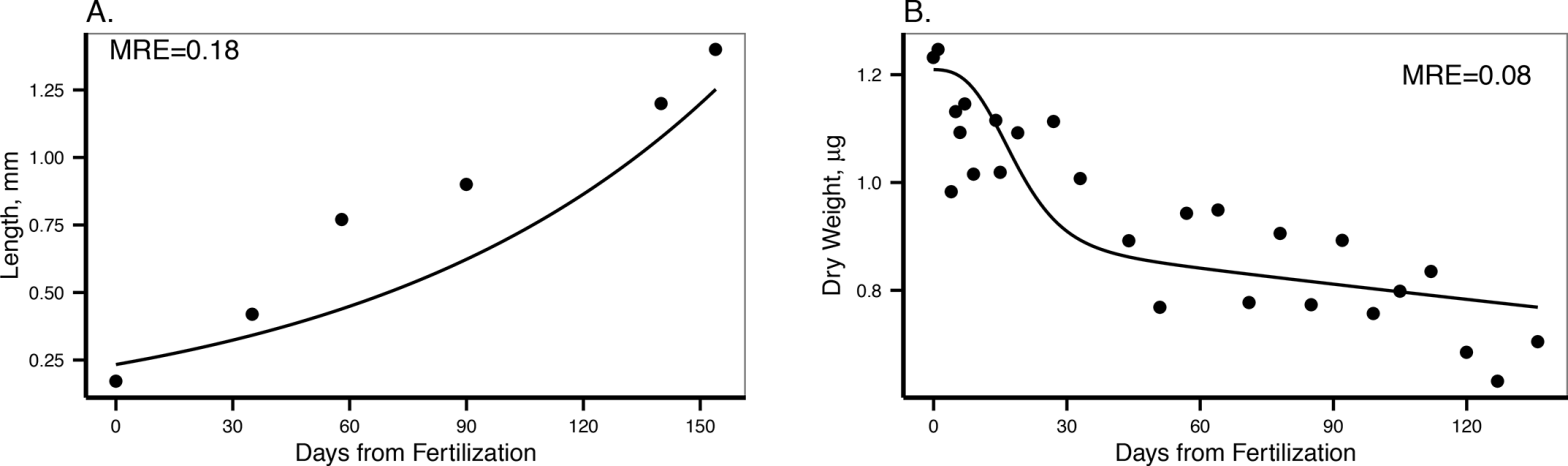
DEB model output with data on pre-metamorphic individuals used for parameter estimation. A. Larval growth in length according to age. Dots are observations in the laboratory [29]. Food was available in excess and the animals fed once they were able to do so. B. Loss of dry weight from fertilization, data from [37]. In this case there was no food available during the whole experiment. MRE is the mean absolute relative error.

The combination of available data from different developmental stages and at different food availabilities allowed us to obtain a robust prediction of DEB parameters. Parameter estimations are detailed in Table 1. The total fit of the covariation method resulted in a MRE (mean absolute relative error) of 0.123. In general, the estimated model parameters accurately describe the data used for their estimation (Table 2, Figs 4, 5 and 6).

The use of individuals originating from the same population (McMurdo Station) in the laboratory and field observations allowed us to approximate the average scaled functional response of *O*. *validus* in the field (Table 1, *f*_*f*_) using the covariation method.

### Reproduction buffer handling rules for gonad development

**Fig 7:**
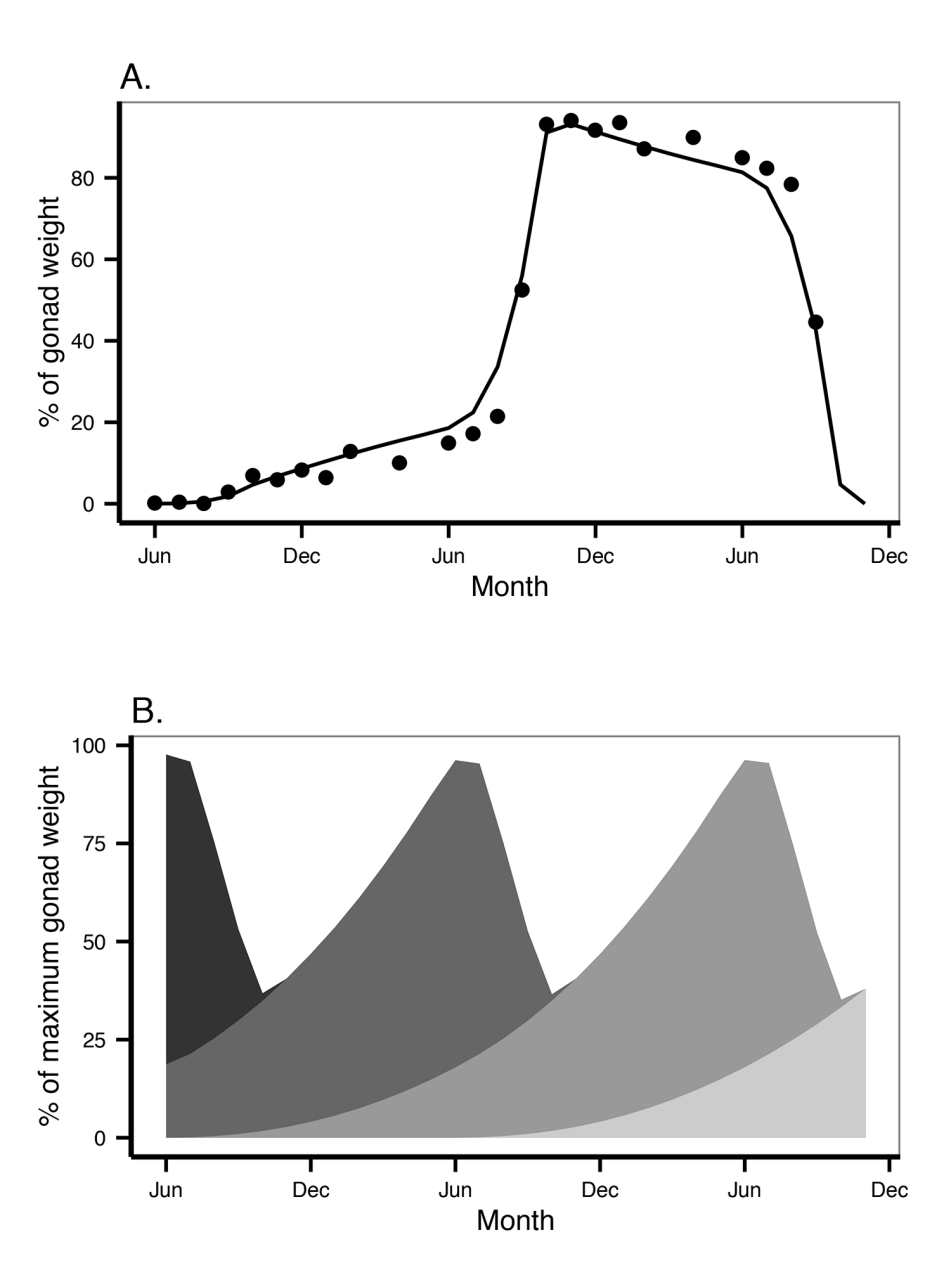
Gametogenesis. A. Proportional weight of one cohort of oocytes as it develop during two years and are released during reproduction. Dots were yielded from field observations made by Pearse [12]. The line is the DEB model output considering *E*_*R*_ handling rules. B. Development of successive cohorts in time, each cohort grows following Eq (3).

Optimization of the rate of cohort growth formulation (3) by non-linear least squares regression yielded a value of *a* = 1.201±0.1 and *b* = 1.157±0.053 (values ± SD). When considering two oocyte cohorts growing simultaneously at different rates (their development started a year apart), the growth rate of the gonad is not constant over time (Fig 7). For example during the reproduction season the gonad is growing slower than ${\dot{p}}_{R}$. However, DEB assumes ${\dot{p}}_{R}$ as continuous and determined by food and body size. On the other extreme, before reproduction takes place, gonads are growing faster than ${\dot{p}}_{R}$. Accordingly, ${\dot{p}}_{R}$ is not immediately transformed into gonad, but accumulated as energy in *E*_*R*_ and transformed into gonads following Eq (3) for each of the cohorts being developed (Figs 2 and 7).

### Model exploration

**Fig 8:**
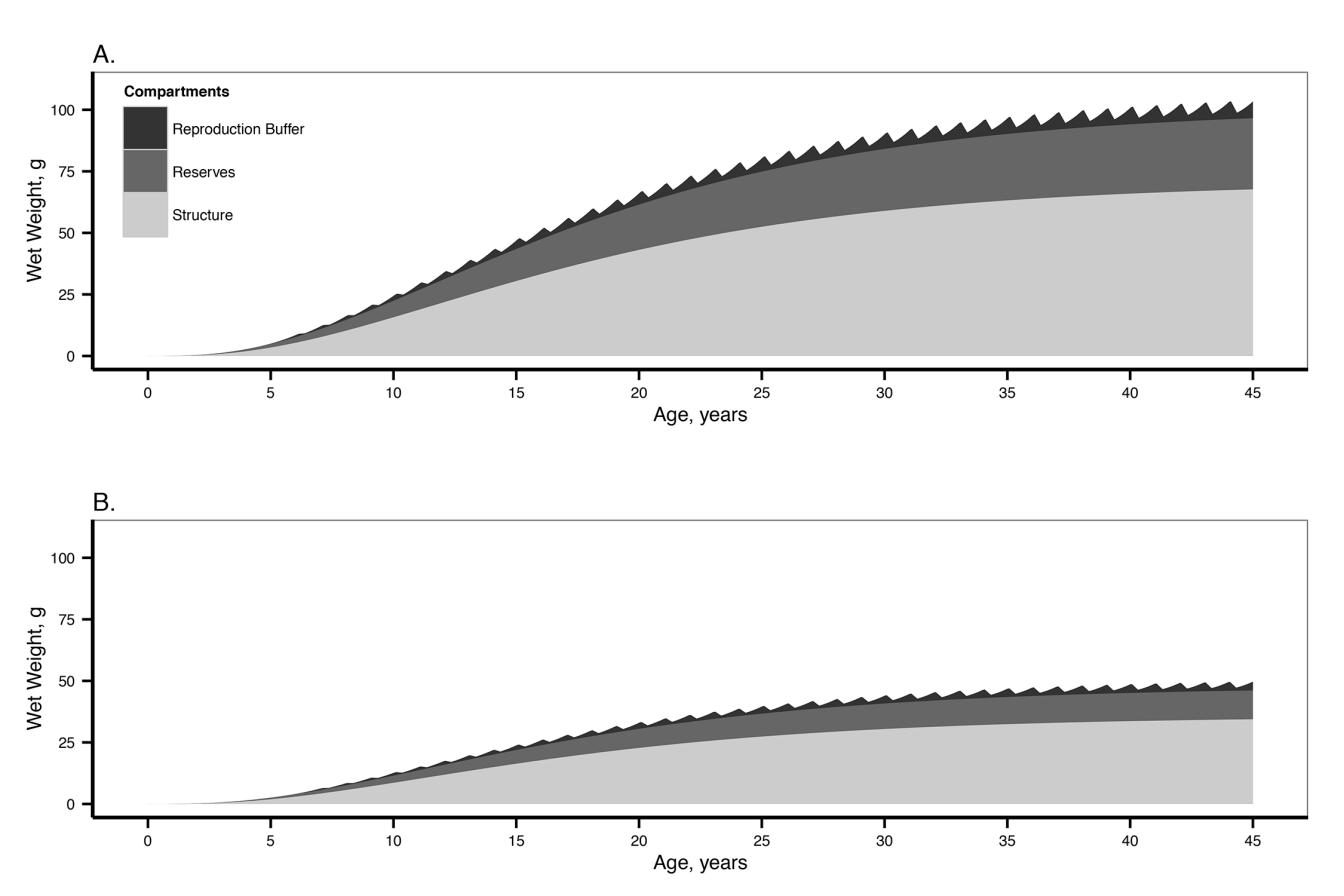
Weight according to age of *O*. *validus*, representing the contributions of the reproduction buffer, the reserves and the structure to the total weight. A. Considering the conditions in the laboratory *ad libitum* feeding. B. Considering the food conditions estimated for McMurdo. For both graphs a constant temperature of 271.5 K was considered.

We explored the model performance by plotting weight from the time of fertilization until reaching the maximum size given by DEB parameters (Fig 8). These plots give an overview of the life history of *O*. *validus* and the differences between the food conditions in the laboratory experiments and the field at McMurdo Station. It takes between 35 and 40 years for *O*. *validus* to reach its maximum size when considering food conditions and temperature being constant during its whole life cycle. They will reproduce for the first time when they are between 5 and 7 years old, and spawn an average of 0.7 to 1.2 million eggs per year. These plots also reveal the importance of the contribution of reserves to the total weight in *O*. *validus*: when fed *ad libitum*, reserves make up to 31% of the total body weight.

**Fig 9:**
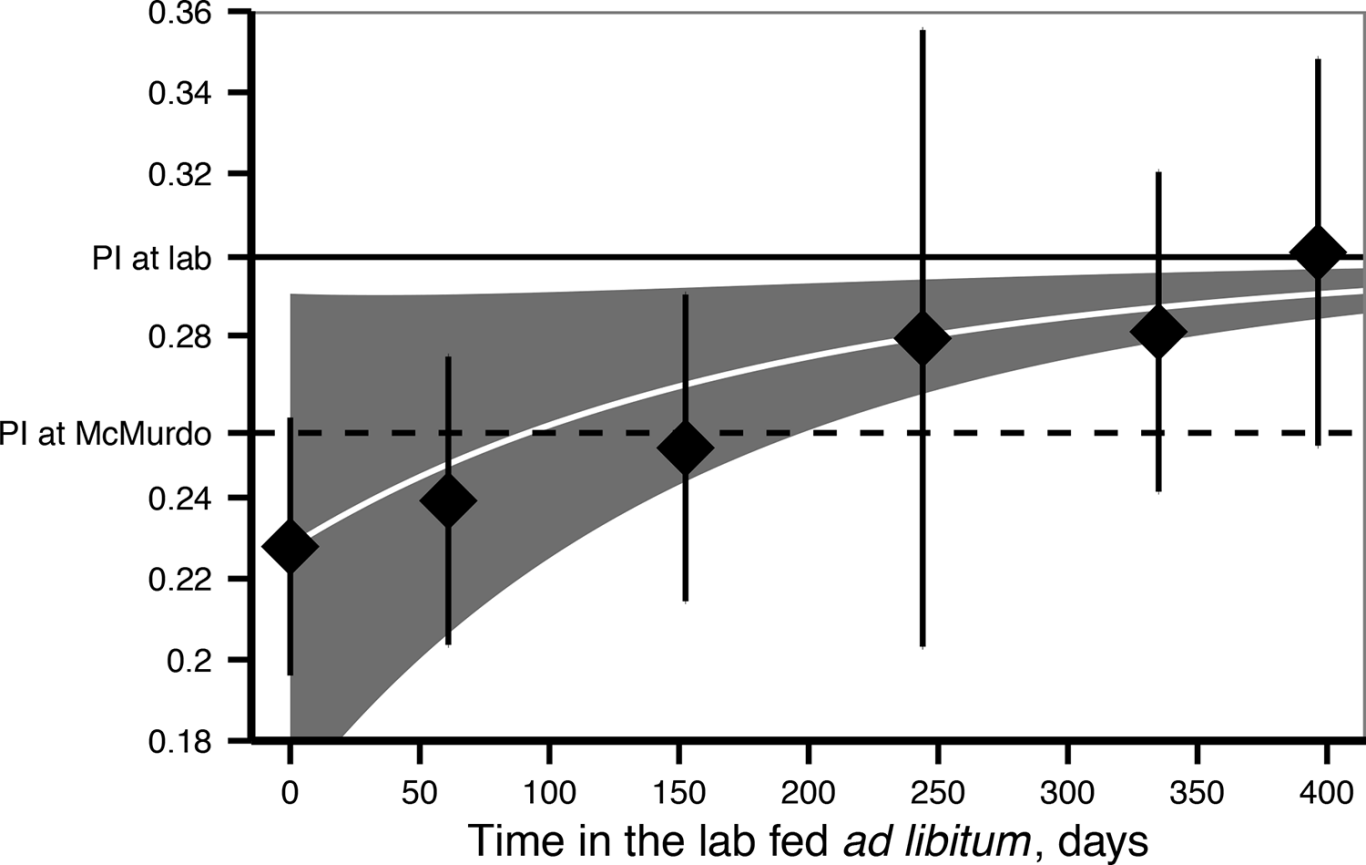
Pyloric Index (pyloric caeca weight / (total weight–gonad weight)) increment after the change in food availability (from the field to the laboratory). Points shows observed PI in the field at the moment of recollection (day 0) and the PI from individuals sampled in the laboratory experiment during the first year. Error bars are standard deviations. White line (mean) and shaded area (95% 95% confidence interval) are DEB model output for the PI, considering the DEB reserve dynamics after a change in food resources (S1 Appendix) applied to the animals used to obtain PI at day 0. Solid line: PI in the lab considering the reserves are in equilibrium with the available food (0.30, *f* = 1). Dashed line: PI for the available food estimated by the model for McMurdo (0.25, *f*_*f*_ = 0.8).

During the parameter estimation, we linked the pyloric caecum of *O*. *validus* to DEB reserves (*E*). Estimations of PI and the pyloric caeca weight in the laboratory after one year of feeding *ad libitum* were accurate (MRE: 0.04, Table 2 and MRE: 0.2, Fig 4 respectively). In the field measurements, PI was not very variable during the year at McMurdo [27]. PI was slightly overestimated for the field samples in October (Fig 9). Predictions and observations of the change in the PI in the laboratory after the change in food availability, revealed that PI increased during the first year, suggesting that the reserves were slowly reaching an equilibrium with the quantity of food available during the experiment (Fig 9) [27].

**Fig 10:**
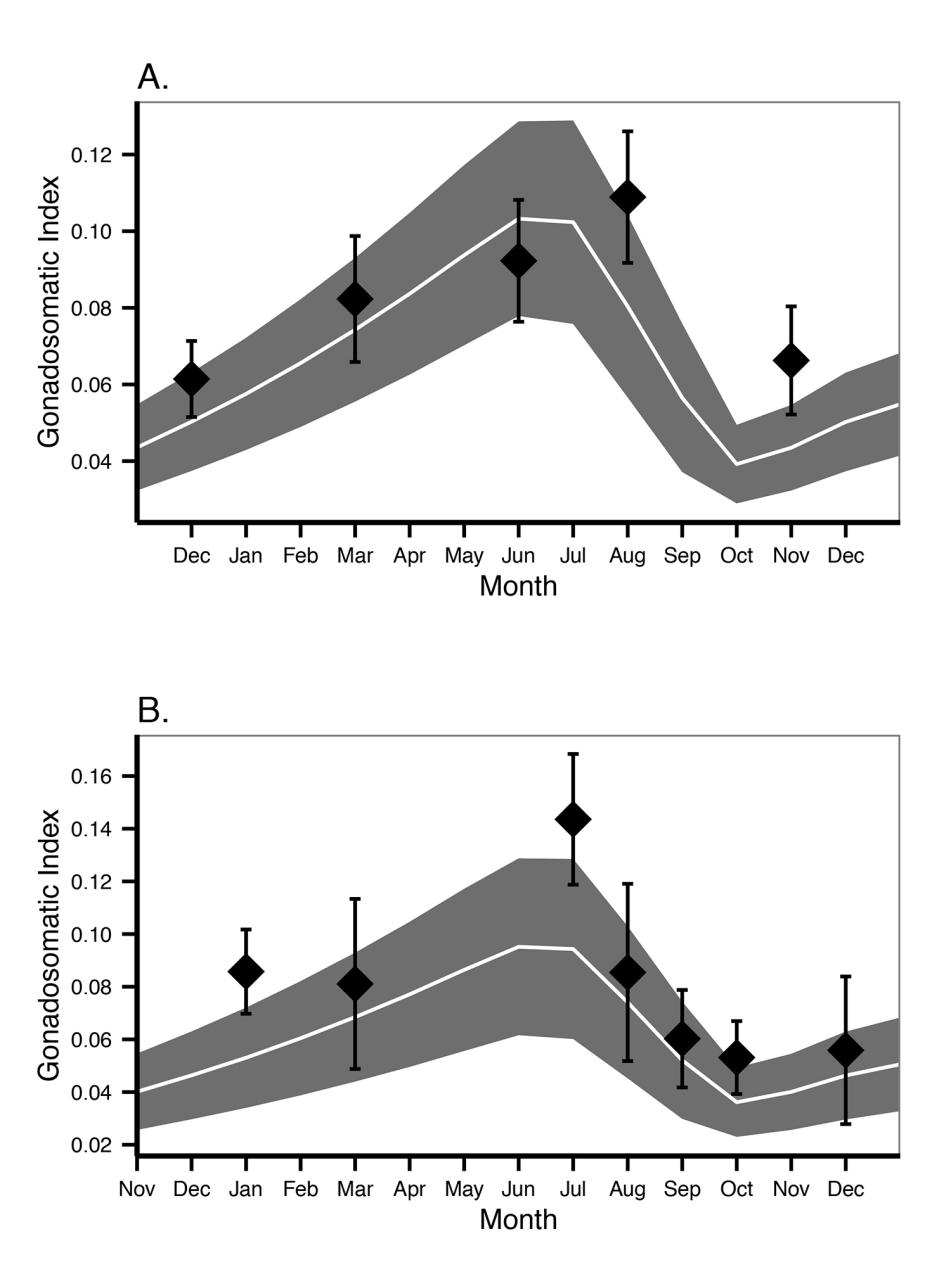
Gonadosomatic Index (GSI). A. Animals kept in the laboratory and fed *ad libitum*. B. Animals at the food level estimated for McMurdo. Points are mean and 95% confidence interval of laboratory and field observations respectively [25]. Line (mean) and shaded areas (95% confidence interval) are DEB model output applying the reproduction buffer handling rules to a 100 individuals with the same mean weight and SD than the ones used in the observations.

The DEB parameters and the reproduction buffer handling rules were also explored by estimating the changes in the gonadosomatic index (GSI) during the year for both cases: in the laboratory and for the field at McMurdo Station (Fig 10). These plots showed that the application of such rules and DEB parameters provided a good proxy to the oogenesis in both cases.

## Discussion

In the present study we successfully estimated the parameters of a DEB model for the sea star *O*. *validus*, an abundant Antarctic marine invertebrate with a key role in shallow benthic communities [47]. The DEB model framework and the parameters estimated herein provide a quantitative eco-physiological model that can be used to understand the physiological conditions of *O*. *validus* and the interactions with its ecosystem. The use of data from the field at McMurdo station allowed us to approximate a scaled functional response for this population, providing insight into the nutritional state of the *O*. *validus* population and the DEB model behaviour when predicting physiological parameters and condition for a wild population. The way this scaled functional response has been calculate mean that it does not relate to an actual resource density. Instead it is a measure of the individuals’ condition related to the difference in their reserves and maximum size to that of the laboratory individuals fed *ad libitum*. The observed difference is related to resource availability and quality at the field, however the lack of information on the functional response of *O*. *validus* makes it impossible to use this parameter to predict actual resource density. In general the values of the parameters respond to a slowed down metabolism. These parameters allowed us to gain insight in the performance of *O*. *validus* under different levels of food resources, and the role of reserves and a low rate of metabolism. Model exploration against laboratory and field observations of GSI and PI provided a first insight of the model’s performance against data independent of that used for model estimation.

### Reserves and varying food availability

DEB parameters highlight how important reserves are for *O*. *validus*. Reserves reached up to 30% of the total body weight in animals fed *ad libitum*. This is also common in other sea stars, where the pyloric caeca usually comprise a significant part of the total body weight [43]. The predicted values during model exploration suggest that the pyloric caeca dynamics can be modelled using DEB parameters. This contrasts with the DEB parameters estimated for the sea star *Pisaster ochraceus*, which yielded significantly smaller reserves [22]. *P*. *ochraceus* also has prominent pyloric caeca, which accounts for up to 20% of its total wet weight in wild populations [48]. Monaco et al. [22] justify their results arguing that the pyloric caeca are located downstream of the DEB reserve compartment, and therefore do not need to be in equilibrium with the environment, which also explains the pyloric cycle [49]. However, the PI reported in the literature is generally calculated as a proportion of the total animal weight [49], and this cycle is in part associated with gonad development and other factors (seasonal variation in food availability and temperature) that may account for seasonal changes in the pyloric caeca and that are directly related to their equilibrium with the food available in the environment [42]. In any case, the PI in *O*. *validus* does not exhibit seasonal cycles when calculated as a proportion of the eviscerated body weight [27]. Predicted PI for animals in the lab was accurate revealing that this organ comprises most of the reserves present. Our results also connected the dynamics of the PI in laboratory animals to that derived from a change in food availability (Fig 9). The change in the PI in the laboratory animals is well explained by the DEB energy reserve dynamics (S1 Appendix). However, predicted PI is still below that of the animals in the lab after a year. This suggests that, although they are rather close, *O*. *validus* reserves are not yet in equilibrium with the food available in the laboratory. As such, both the PI and body size of *O*. *validus* may grow beyond the maximum observed after one year. Unfortunately no data on the second year of the experiment performed by Pearse and Bosch [27] is available to confirm this possibility. Further research is needed to link the dynamics of the pyloric caeca to DEB as this may be used as a tool to understand the effects of environmental change on individual fitness and population dynamics of *O*. *validus* and other sea stars.

The DEB parameters estimated for *O*. *validus* indicate a slowed down metabolism, with a very low somatic maintenance [${\dot{p}}_{M}$] and a slow growth and maturation. [${\dot{p}}_{M}$] is not very different from that of other sea stars (*Asterias rubens* and *Asterina gibbosa*, DEBlab^2^) that are between 8–9 J cm^-3^ d^-1^ at 285K (an exception is *P*. *ochraceus* which [${\dot{p}}_{M}$] is about 27 J cm^-3^ d^-1^). The somatic maintenance [${\dot{p}}_{M}$] of *O*. *validus* at the temperature at which it normally lives (around 0°C) is barely of 3 J cm^-3^ d^-1^. *O*. *validus* experiences an environment where food is extremely variable spatially and temporally, experiencing long periods of food shortage or consuming energetically poor food items [5,13]. The combination of an important energy reserve and low metabolic costs theoretically allows a fully fed individual to undergo up to a year of starvation before running out of reserves (energy reserves/maintenance costs). This is an important adaptation of *O*. *validus* to its environment. However the capacity to withstand starvation is reduced as temperature increases. Other sea stars such as *Pisaster ochraceus* and *Asterias rubens* have the capacity of using their body wall as an energy source [42,50] reducing the energy demands of the body wall and the need to produce energy to maintain it. There is no information on such a capacity for *O*. *validus*. Under field conditions where reserves are reduced a capacity to use the body wall as an energy source, or even the gonads, may be necessary for survival. Energy conductance ($\dot{\nu }$), which determines the mobilization from reserves (S1 Appendix), is larger in *O*. *validus* than in the same related species ($\dot{\nu }$ = 0.004±0.001 cm d^-1^), which suggests that *O*. *validus* can maintain a larger mobilization flux (${\dot{p}}_{C}$) at low temperatures, an important adaptation necessary to its habitat.

### Reproduction

*Odontaster validus* is a broadcaster spawner that releases its gametes once a year. Oogenesis, however, is a longer process that takes from 18 and 24 months [12,26]. This mechanism involves the simultaneous development of two different size cohorts of oocytes, and is a strategy displayed by several species of sea stars in the Southern Ocean, as well as other echinoderms [51,52], crustaceans [13], and annelids [14]. The fraction of energy allocated to somatic maintenance and growth (*κ*) is greater in *O*. *validus* than in other sea stars and other echinoderms (*κ* = 0.6±0.1; DEBlab) (http://www.bio.vu.nl/thb/deb/deblab/add_my_pet_old/Species.html), which implies a smaller energy investment in reproduction. This reduced investment coupled to a sluggish metabolism suggests, a priori, a smaller reproductive output than echinoderms from temperate regions. However, although GSI is lower, annual reproductive output is not very different from that of other sea stars [53–55]. Developing two oocyte cohorts simultaneously, and in a continuous way means that a fraction of the reproduction buffer is always present in the organism (at minimum between 20 to 40% of it). For other sea stars, somatic maintenance has a priority over reproduction [41,42]. The energy present in the form of gonads may be an extra energy resource in case of prolonged starvation [22,41]. Alternatively, in case it is not used, it could serve to satisfy an emergency reproduction without further energy investment [56]. Whether the capacity to maintain a high fecundity is a consequence of the oogenesis strategy or only related to the low maintenance costs and increase conductivity could not be disentangled by the data currently available. However the model suggests that the parameters estimated here are able to predict gonad development and reproductive output and therefore that success is linked to energy handling and environmental conditions.

The reproduction buffer handling rules estimated here allowed us to predict the GSI changes during the year and how the oogenesis of simultaneous oocyte cohorts takes place in the context of energy investment in reproduction. These rules allowed us to understand that although oogenesis is a constant process it still has a strong seasonal variability, as has been observed previously [12]. Oogenesis is less intense during spawning and increases as a power function during the year being maximal just before the reproduction season. *O*. *validus* reproduces during winter, to ensure the feeding larvae are present by spring when algae blooms start [26]. Winter is also when food may be more scarce for adults [12] and therefore there may be less energy available to invest in reproduction. It is noteworthy that while the data used to estimate the handling rules is based on field data, the DEB model assumes a constant condition of food and temperature (averaged) during the whole oogenesis, which means that the variability in the oogenesis process may be a reflection of the environmental conditions. However more data on the role of food availability and the oogenesis process is necessary to disentangle these effects. The onset of development for each oocyte cohort is set June in the current model, reflecting the case of the population in McMurdo [12]. However gametogenesis and reproduction seem to be controlled by photoperiod [27] and as such reproduction periodicity will change between locations. This factor can be included in future DEB model application. There is no data with the same level of detail to fully model the spermatogenesis process, however GSI values does not differ significantly between *O*. *validus* males and females [12,43,57].

The Southern Ocean includes areas experiencing a rapid warming [6,7]. The changes in the environment resulting from global change will have an important effect on the biodiversity and ecology of the Southern Ocean, affecting species distribution and population dynamics [58,59]. This results in a pressing need to gain knowledge on how the performances of Southern Ocean organisms are affected and to predict how future global change scenarios will impact their distribution and abundance [10]. There has been an important effort to try to predict Southern Ocean species distributions and link them to the environmental conditions. These species distribution models (SDMs) were generally based on the principle of the fundamental niche and focused on statistical correlations between environmental variables and knowledge of current distributional patterns [60]. Those models do not include any aspect related to the organism biology, moreover our knowledge on the current distribution of Southern Ocean species is still limited [11]. However, how the organisms respond to the environmental changes resulting from global warming and their ability to cope with them is determined by their metabolic processes [61]. It is necessary to incorporate the biology and ecophysiology of the organisms into their distribution models through mechanistic SDMs [60]. DEB theory is able to explain the whole life cycle of an organism as a function of constant parameters. DEB models are well suited for their application in mechanistic species distribution models (SDM) [61] allowing us to integrate the organism’s ecophysiology with the changes observed in the physical environment. Moreover these parameters allow the prediction of the effect of environmental changes on different stages using a theoretical energy-based framework. In this study we estimated DEB parameters from available data in the literature. The large amount of research on the physiology of several species from the Southern Ocean can be used to rapidly advance the integration of the DEB framework with the need to predict the fate of Antarctic biodiversity under climate change. Further experiments and data acquisition will still be necessary. DEB theory is a basic general framework and the DEB model provides opportunities to incorporate additional mechanisms that may have an effect on the metabolic processes. In that manner it should be possible to add to the standard DEB model the effects of other stressors on the DEB parameters, such as ocean acidification [62] or salinity [63].

## Conclusion

The model developed here for *O*. *validus* allowed us to increase our knowledge on the ecophysiology of this species, providing us new insights on the role of food availability and temperature on its life cycle and reproduction strategy. The DEB model adds some insights in the adaptations to its particular habitat, such as the capacity to mobilize energy faster than temperate sea stars or the ability to withstand long starvation periods thanks to its massive reserves. The DEB model also provides information to fill some existing gaps in *O*. *validus* physiology and biology. Early growth from metamorphosis can now be modeled using DEB parameters. The DEB model shows that *O*. *validus* is a slow growing species, taking at least 35 years to reach its maximum size, reproducing for the first time when its 7 years old, and exhibiting a relatively high fecundity. This DEB model gives an insight on the role of food resources and temperature on the physiology *O*. *validus*, although further research on this species functional response is needed. Temperature limits obtained in this study must also be taken with care, temperature sensitivity is based on a short term experiment [29] and it is necessary to further explore which temperatures can *O*. *validus* can stand permanently. Development of reproduction buffer handling rules allowed us to further explain allocation of energy to gonads during oogenesis and the development of simultaneous cohorts. The link presented here for the first time between the DEB model reserves and a body compartment provides a mechanistic tool for direct observation of the effects of environmental change on individual fitness. These findings yielded through the present DEB model are of importance for the understanding of *O*. *validus* population dynamics and the effects on it of environmental changes associated to climate change, presenting the DEB model as a useful tool for conservation planning.

## Supporting Information

S1 Appendix General description of standard DEB model assumptions and notation. (DOCX)
